# Supplementary material for: Concerted transformation of a hyper-paused transcription complex and its reinforcing protein
Source: Nat Commun. 2024 Apr 8;15:3040. doi: 10.1038/s41467-024-47368-4 (PMC11001881; doi:10.1038/s41467-024-47368-4)
Supplement: Supplementary file 1 — Supplementary Information [file 41467_2024_47368_MOESM1_ESM.pdf]

Supplementary Information

Concerted transformation of a hyper-paused transcription complex and its reinforcing protein

Philipp K. Zuber[1,#], Nelly Said[2], Tarek Hilal[2,3], Bing Wang[4], Bernhard Loll[2,3], Jorge González-Higueras[5,6], César A. Ramírez-Sarmiento[5,6], Georgiy A. Belogurov[7], Irina Artsimovitch[4,*], Markus C. Wahl[2,8,*], and Stefan Knauer[1,&,*]

[1] Universität Bayreuth, Biochemistry IV - Biophysical Chemistry, Bayreuth, Germany

[2] Freie Universität Berlin, Institute of Chemistry and Biochemistry, Laboratory of Structural Biochemistry, Berlin, Germany

[3] Freie Universität Berlin, Research Center of Electron Microscopy and Core Facility BioSupraMol, Germany

[4] The Ohio State University, Department of Microbiology and Center for RNA Biology, Columbus, OH, USA

[5] Pontificia Universidad Católica de Chile, Institute for Biological and Medical Engineering, Schools of Engineering, Medicine and Biological Sciences, Santiago, Chile

[6] ANID, Millennium Science Initiative Program, Millennium Institute for Integrative Biology, Santiago, Chile

[7] University of Turku, Department of Life Technologies, Turku, Finland

[8] Helmholtz-Zentrum Berlin für Materialien und Energie, Macromolecular Crystallography, Berlin, Germany

[#] Current address: MRC Laboratory of Molecular Biology, Cambridge Biomedical Campus, Cambridge, United Kingdom

[&] Current address: Bristol-Myers Squibb GmbH & Co., Munich, Germany

* Correspondence to: artsimovitch.1@osu.edu, markus.wahl@fu-berlin.de, Stefan.knauer@bms.com

Supplementary Discussion

Sequence-structure relationships in PECs

Comparison of *ops*PEC with structurally-characterized PECs, *his*PEC[1,2], *his*-ePEC[3] and con-ePEC[3], offers insights into the mechanism of pausing and the contributions of the individual sequence determinants. Con-ePEC is paused on a synthetic sequence GG-CAUAGUUG-CG based on the pause consensus ($^{-12}$GG-NNNNNNNN-YG^{+1})[4,5]; GG and YG motifs are known as upstream and downstream fork junction (UFJ and DFJ) determinants, respectively. The consensus within the 8N region is weak and, in the first approximation, the con-ePEC structure reveals contributions of DFJ and UFJ to pausing.

*ops*PEC (GG-CGGUAGCG-UG) and *his*PEC (upstream RNA hairpin followed by CG-AUGUGUGC-UG) form on naturally occurring regulatory pause elements featuring nucleic acid hairpins. In *ops*PEC, the hairpin forms in the NT DNA and is encoded by the 8N sequence, and perfect DFJ and UFJ are present. In *his*PEC, a nascent RNA hairpin forms in the exit channel. *his*PEC and *his*-ePEC, its hairpin-less derivative, have perfect DFJ and a partial UFJ.

The common feature of classic factor-independent PECs is failure to translocate and load the acceptor T-DNA base into the active site. *ops*PEC and con-ePEC are pre-translocated, *his*-ePEC equilibrates between pre- and half-translocated states (only RNA translocates), and hisPEC is half-translocated. The asynchronous (half) translocation leads to tilting of the nucleobases of the RNA:DNA hybrid relative to the helical axis. Since incomplete translocation is characteristic for all these pauses, the DFJ and UFJ are likely mainly responsible for the translocation block.

The synthetic con-ePEC and *his*-ePEC equilibrate between several states with open and closed active sites[3]. In contrast, natural *ops*PEC and *his*PEC are each represented by a single state with an open active site, likely because both are strongly stabilized in a swiveled conformation by nucleic acid hairpins, and swiveling is incompatible with the folded TL[1]. Noteworthy, a fraction of *his*-ePEC is also swiveled despite lacking the stabilizing hairpin. The causative relationships between hybrid tilting and swiveling were hitherto difficult to delineate.

Considering that *ops*PEC is swiveled but not tilted suggests that tilting of hisPEC/ePEC is mediated by the 8N sequence. Indeed, AUGUGUGC consists entirely of purine-pyrimidine steps, has diminished intra-strand base stacking, and is expected to favor tilting of nucleobases to enhance the inter-strand stacking of purines. Interestingly, while both *ops*PEC and *his*PEC are swiveled, the swiveling is seemingly attributable not to the UFJ and DFJ determinants, but to pause-specific 8N regions, the *ops*HP and the tilted hybrid, respectively. In the latter case, swiveling is further stabilized by the RNA hairpin.

A notable feature of the con-ePEC is an overextended 11-bp RNA:DNA hybrid, which forms only upon active site opening and is not linked to swiveling – all states of con-ePEC are not swiveled. *ops*PEC also forms an 11-bp hybrid, but only upon RfaH binding. It is tempting to speculate that *ops*HP inhibits the formation of the overextended hybrid, whereas RfaH binding repositions the *ops*HP and unleashes the intrinsic potential of *ops*PEC to form such a state. Hybrid overextension is a step backward and may conceivably delay translocation, thereby strengthening the pause, but the underlying sequence determinants are difficult to pinpoint unambiguously. One possibility is that the DFJ and UFJ determinants are sufficient to cause hybrid overextension, and the inability of *his*PEC/ePEC to form such a state can then be attributed to hybrid tilting, the partial UFJ, or both. Another possibility is that specific determinants within the 8N sequence are the reason for hybrid overextension: con-ePEC and *ops*PEC, but not *his*PEC/ePEC, share G at -2 and C at -9.

Supplementary Tables

Supplementary Table 1. CryoEM data collection, refinement, and validation statistics for complexes assembled on the *ops*PEC scaffold

	***ops*PEC** (PDB 8PDY) (EMDB 17626)	***ops*PEC^{Enc}** (PDB 8PIB) (EMDB 17679)	***ops*PEC^{Rec} State 1** (PDB 8PHK) (EMDB 17668)	***ops*PEC^{Rec} State 2** (PDB 8PEN) (EMDB 17632)	***ops*PEC^{Back}** (PDB 8PID) (EMDB 17681)	***ops*PEC^{Rec} + NusA** (PDB 8PIL) (EMDB 17685)
**Data collection and processing**						
Microscope	FEI Titan Krios G3i					
Voltage [keV]	300					
Camera	Falcon 3EC					
Magnification (nominal/calibrated)	96,000	96,000	96,000		96,000	96,000
Pixel size at detector [Å/pixel]	0.832	0.832	0.832		0.832	0.832
Total electron exposure [e^{-}/Å^2]	42	42	42		42	42
Exposure rate [e^{-}/pixel/s]	0.7	0.7	0.7		0.7	0.7
Frames collected during exposure [no.]	33					
Defocus range [μm]	0.80 - 2					
Automation software	EPU (version 2.8.1)					
Micrographs collected [no.]	3,790	5,094	3,382		3,341	4,226
Micrographs used [no.]	3,613	4,986	2949		2,823	3,142
Total extracted particles [no.]	759,854	1,090,135	1,040,421		360,161	877,027
Final particles [no.]	250,888	848,008	124,233		297,862	41,797
Point-group or helical symmetry parameters	C1	C1	C1		C1	C1
Resolution (global) [Å] FSC 0.143 (unmasked/masked)	4 / 3.5	3.2 / 2.6	3.7 / 3.1		3.5 / 3	4 / 3.2
Resolution range (local) [Å]	2.8 – 30.00	1.95 – 30.00	2.5 – 30.00		2.2 – 30.00	2.4 – 30.00
Map sharpening *B* factor [Å^2] / (*B* factor range)	-130	-85	-95		-100	-78
Map sharpening methods	local B-factor					
Refinement package	PHENIX (version 1.20_44591) real.space.refine					
**Model composition**						
Non-hydrogen atoms	26,887	27,850	28,225	27,734	28,297	30,440
Protein residues	3,221	3,316	3,370	3309	3,371	3,656
DNA nucleotides	70	80	76	76	76	72

RNA nucleotides	12	12	12	12	14	12
Mg^{2+} ions	1	1	1	1	1	1
Zn^{2+} ions	2	1	2	2	2	2
**Model refinement**						
Model-Map scores						
CC (mask)	0.87	0.88	0.86	0.87	0.88	0.84
CC (volume)	0.86	0.87	0.85	0.86	0.87	0.82
Average grouped B factors [Å^2]						
Overall	161	116	125	141	94	121
Protein residues	159	111	116	135	93	120
DNA nucleotides	198	200	280	179	113	140
RNA nucleotides	154	78	133	110	97	104
Mg^{2+} ion	154	104	97	150	81	133
Zn^{2+} ions	135	127	137	171	106	131
RMSD from ideal values						
Bond lengths [Å]	0.002	0.003	0.002	0.002	0.002	0.002
Bond angles [°]	0.547	0.568	0.517	0.548	0.477	0.484
**Validation**						
MolProbity score	1.68	2.17	1.59	1.70	1.49	1.60
CaBLAM outliers [%]	1.76	1.95	1.80	1.65	2.04	2.02
Clashscore	10.44	9.35	9.15	9.32	7.35	9.19
Poor rotamers [%]	0.22	2.6	0.1	1.5	0.3	0.2
Cβ deviations	0	0	0	0	0	0
EMRinger score	1.41	1.85	1.19	1.55	1.58	1.58
Ramachandran plot						
Favored [%]	97.2	97.3	97.5	97.6	97.6	97.4
Allowed [%]	2.8	2.6	2.5	2.3	2.4	2.6
Outliers [%]	0.0	0.1	0.0	0.0	0.0	0.0
Ramachandran plot *Z*-score (RMSD)						
Whole	0.68 (0.15)	0.93 (0.15)	1.00 (0.15)	1.05 (0.15)	1.18 (0.15)	1.07 (0.14)
Helix	1.68 (0.16)	1.98 (0.16)	2.07 (0.16)	2.05 (0.16)	2.38 (0.16)	2.08 (0.15)
Sheet	0.11 (0.25)	0.52 (0.24)	0.24 (0.23)	0.38 (0.23)	0.38 (0.24)	0.32 (0.23)
Loop	-0.28 (0.15)	-0.33 (0.15)	-0.16 (0.15)	-0.16 (0.15)	-0.16 (0.15)	-0.08 (0.15)

Supplementary Table 2. CryoEM data collection, refinement, and validation statistics for complexes assembled on the nc-*ops*PEC scaffold

	**nc-*ops*PEC**<br><br>(PDB 8PH9)<br>(EMDB 17657)	**nc-*ops*PEC^{Enc}**<br><br>(PDB 8PFG)<br>(EMDB 17646)	**nc-*ops*PEC^{Rec} State 1**<br>(PDB 8PIM)<br>(EMDB 17686)	**nc-*ops*PEC^{Rec} State 2**<br>(PDB 8PFJ)<br>(EMDB 17647)
**Data collection and processing**				
Microscope	FEI Titan Krios G3i			
Voltage [keV]	300			
Camera	Falcon 3EC			
Magnification (nominal/calibrated)	96,000	96,000	96,000	
Pixel size at detector [Å/pixel]	0.832	0.832	0.832	
Total electron exposure [e^{-}/Å^2]	42	42	42	
Exposure rate [e^{-}/pixel/s]	0.7	0.7	0.7	
Frames collected during exposure [no.]	33			
Defocus range [µm]	0.60 - 2			
Automation software	EPU (version 2.8.1)			
Micrographs collected [no.]	4,332	5,230	4,614	
Micrographs used [no.]	4,261	4,979	4,449	
Total extracted particles [no.]	510,420	1,519,652	849,514	
Final particles [no.]	242,435	509,867	51,791	
Point-group or helical symmetry parameters	C1	C1	C1	
Resolution (global) [Å] FSC 0.143 (unmasked/masked)	3.5 / 3	3.5 / 3.1	4 / 3.4	
Resolution range (local) [Å]	2.54 – 30.00	2.48 – 30.00	2.8 – 30.00	
Map sharpening *B* factor [Å^2] / *B* factor range	-90	-96	-73	
Map sharpening method	local B-factor			
Refinement package	PHENIX (version 1.20_44591) real.space.refine			
**Model composition**				
Non-hydrogen atoms	26,957	27,809	28,232	27,856
Protein residues	3,218	3,311	3,374	3,313
DNA nucleotides	72	80	70	76
RNA nucleotides	15	12	17	17
Mg^{2+} ions	1	1	1	1
Zn^{2+} ions	2	1	2	2

**Model refinement**				
Model-Map scores				
CC (mask)	0.90	0.88	0.87	0.86
CC (volume)	0.89	0.87	0.86	0.86
Average grouped B factors [Å^2]				
Overall	116	121	151	153
Protein residues	111	116	147	146
DNA nucleotides	185	208	184	271
RNA nucleotides	178	115	287	112
Mg^{2+} ion	101	135	152	149
Zn^{2+} ions	168	113	199	177
RMSD from ideal values				
Bond lengths [Å]	0.002	0.003	0.007	0.002
Bond angles [°]	0.476	0.449	0.657	0.536
**Validation**				
MolProbity score	1.58	2.21	1.92	1.94
CaBLAM outliers [%]	1.57	1.90	1.83	1.98
Clashscore	7.61	9.04	9.2	9.27
Poor rotamers [%]	1.5	5.5	2.7	3.15
Cβ deviations	0	0	0	0
EMRinger score	2.02	1.92	1.28	1.50
Ramachandran plot				
Favored [%]	97.8	97.1	97.5	97.7
Allowed [%]	2.2	2.9	2.5	2.3
Outliers [%]	0.0	0.0	0.0	0.0
Ramachandran plot *Z*-score (RMSD)				
Whole	1.39 (0.15)	0.76 (0.15)	1.21 (0.15)	1.51 (0.15)
Helix	2.36 (0.16)	1.82 (0.15)	2.30 (0.16)	2.29 (0.16)
Sheet	0.66 (0.24)	-0.01 (0.24)	0.71 (0.24)	0.72 (0.24)
Loop	0.03 (0.15)	-0.28 (0.16)	-0.20 (0.15)	0.23 (0.16)

Supplementary Table 3. Oligonucleotides and recombinant DNA

Oligonucleotides	Source	Identifier
RNA (*ops*PEC):<br>5’-UCU AUA UGU CAG CGU GU-3’	Metabion	-
RNA (nc-opsPEC):<br>5’-UUC UUU GGC GGU AGC GU-3’	Metabion	-
T-DNA (*ops*PEC):<br>5’-GGA AGA TCG AAA AAA GCA CAC GCT GAC CCG CGT GGT GGT G-3’	Metabion	-
T-DNA (nc-*ops*PEC):<br>5’-GGA AGA TCG AAA AAA GCA CGC TAC CGC CCG CGT GGT GGT G-3’	Metabion	-
NT-DNA:<br>5’-CAC CAC CAC GCG GGC GGT AGC GTG CTT TTT TCG ATC TTC C-3’	Metabion	-
Transcription template (forward):<br>5’-GGAGAGACAACTTAAAGAGACTT-3’	Millipore Sigma	Lab stock #1923
Transcription template (reverse):<br>5’-GGAAGATGATCTTCCGGGGGCTT-3’	Millipore Sigma	Lab stock #1112
**Recombinant DNA**	**Source [6]**	**Identifier**
The *ops*P transcription template:<br>T7A1-*ops*P-*his*P	Supplementary Reference [6]	pIA349
The *ops*PEC transcription template:<br>T7A1-G38-*ops*P-*his*P	This paper	pIA1633
RNAP expression vector (wt):<br>T7P–α–β–β'-His$_6$–ω	Supplementary Reference [7]	pVS10
RNAP expression vector:<br>T7P–α–His$_6$-β[R371A, R394A]–β'–ω	This paper	pIA1427
RNAP expression vector:<br>T7P–α–His$_6$-β[R470A, R473A]–β'–ω	This paper	pIA1428
RNAP expression vector:<br>T7P–α–His$_6$-β[R542A]–β'–ω	This paper	pIA1429
RNAP expression vector:<br>T7P–α–β–β'[R314A]-His$_6$–ω	This paper	pIA1435
σ^{70} expression vector:<br>T7P–His$_6$-σ^{70}	Supplementary Reference [8]	pIA586
RfaH expression vector:<br>T7P– His$_6$–TEV–RfaH	Supplementary Reference [9]	pIA751
RfaH-NGN expression vector:<br>T7P–RfaH-NGN–TEV–KOW-His$_6$	Supplementary Reference [9]	pIA750
GreA expression vector:<br>T7P–GreA-His$_6$	This paper	pIA578
Mfd expression vector	Supplementary Reference [10]	pAD6
NusA expression vector	Supplementary Reference [11]	pTKK19 *nusA*(1-495)
RfaH expression vector	Supplementary Reference [12]	pET19bmod_*rfaH*
RfaH^{CC} expression vector	This paper	pET19bmod_*rfaH*$^{F51C;S139C}$

Supplementary Table 4. Summary of simulation systems

Simulation system	Simulation box dimensions [nm]	Total number of atoms	Total number of water molecules	Total number of ions	Box geometry
*ops*PEC^{Rec} + active RfaH	18.3 × 18.3 × 18.3	607,567	183,647	155 Na^{+}<br>2 Zn^{+2}	Cubic
*ops*PEC^{Rec} + autoinhibited RfaH	19.5 × 19.5 × 19.5	734,641	226,005	155 Na^{+}<br>2 Zn^{+2}	Cubic
Dual-basin SBM *ops*PEC^{Rec} + RfaH	-	28,484	-	2 Zn^{+2}	-
Dual-basin SBM RfaH	-	1,295	-	-	-

Supplementary Figures

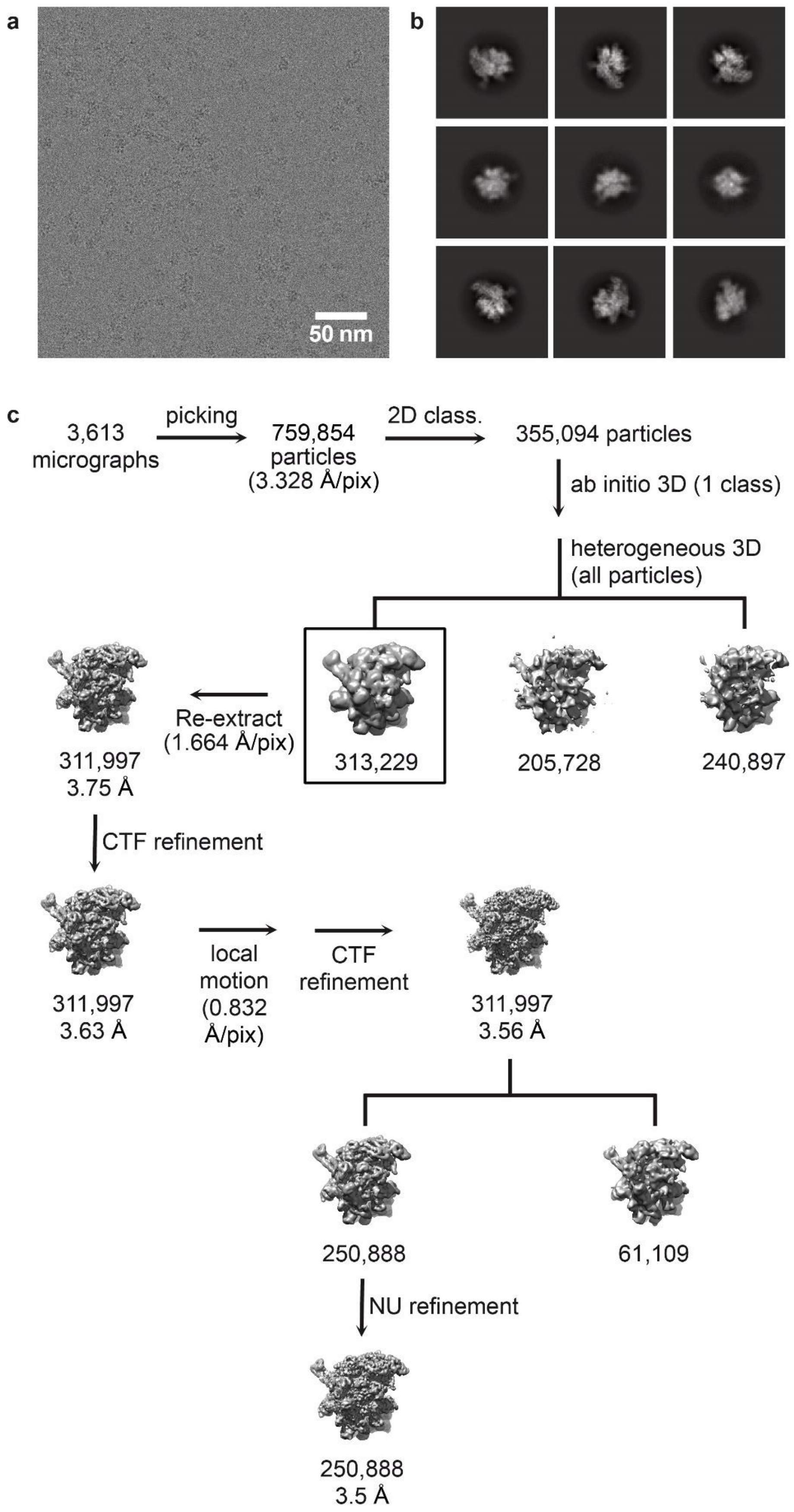

Supplementary Figure 1. CryoEM data acquisition and processing. a-c, Representative micrograph (**a**), 2D class averages (**b**) and data processing flowchart (**c**) of the *ops*PEC cryoEM data acquisition and processing. The micrographs, 2D classes and workflow are highly similar for the samples of all presented structures and thus serve as representative example. The scale bar in (**a**) represents 50 nm. Class averages in (**b**) were generated using reference-free 2D classification within cryoSPARC. **c**, A total of 3,613 aligned movies were selected for further analysis. Initially picked particles (759,854) were extracted fourier-cropped to a pixel size of 3.328 Å and subjected to reference-free 2D classification. Shiny 2D class averages were selected for ab initio 3D reconstruction to generate an initial reference for heterogeneous 3D refinement of the whole dataset. The best appearing class (boxed) was re-extracted with reduced fourier cropping and subjected to homogeneous and CTF refinement before local motion correction was conducted. Final particle images were selected by 3D heterogeneous refinement after another cycle of homogeneous and CTF refinement. Non-uniform refinement of 250,888 selected particle images resulted in a final reconstruction at a global resolution of 3.5 Å.

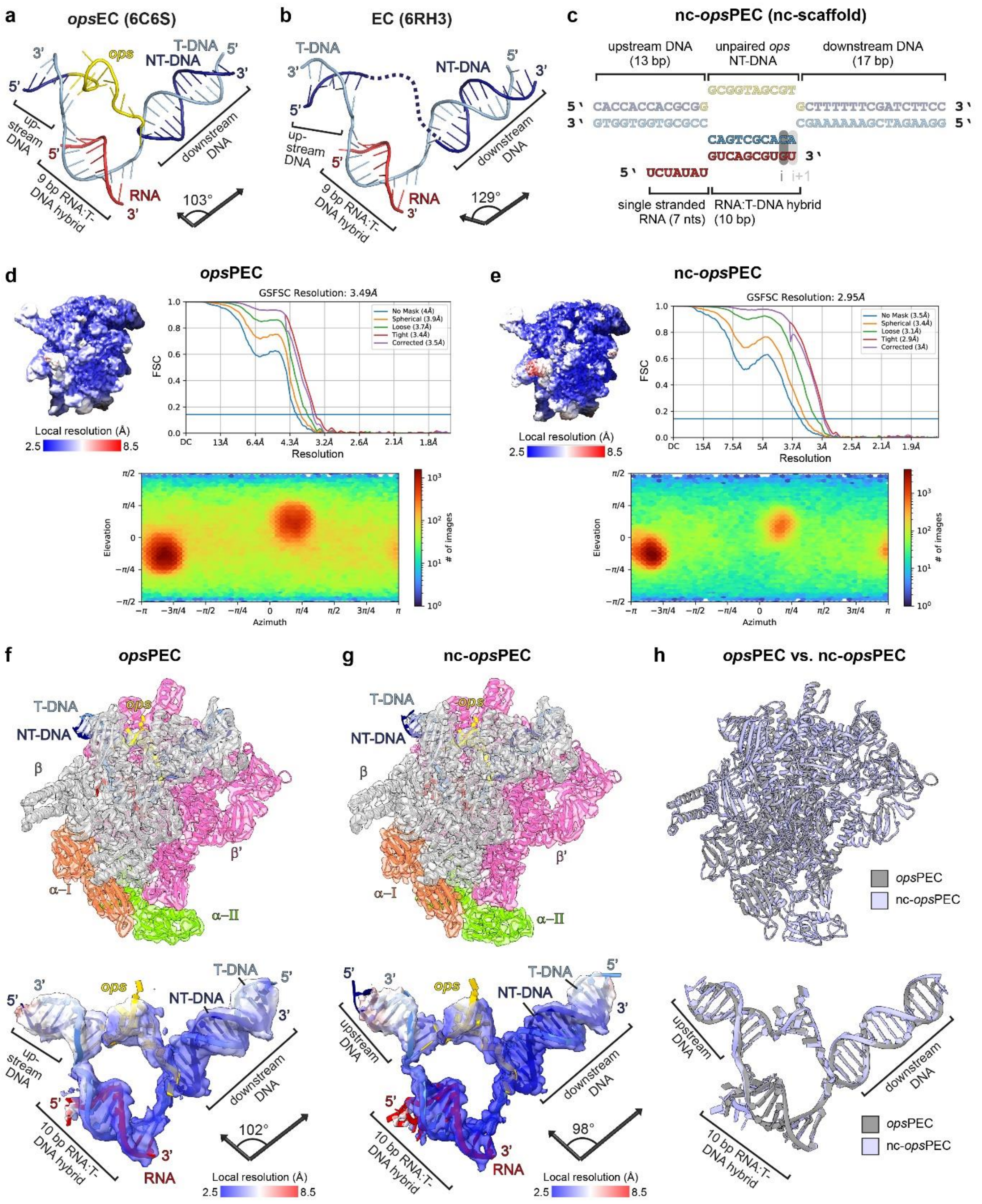

Supplementary Figure 2. Structural comparison of the *ops*PEC with other ECs and the nc-*ops*PEC. a,b, Nucleic acid scaffolds of the *ops*EC (**a**; PDB-ID: 6C6S) and a pre-translocated EC (**b**; PDB-ID: 6RH3) in cartoon representation. Helix vectors of the up- and downstream DNA (labelled) and angle between them are shown. **c**, Nucleic acid scaffold used for reconstitution of the pre-translocated nc-*ops*PEC, (harboring a non-complementary

transcription bubble). Regions identical to the scaffold of *ops*PEC (Fig. 1b) are grayed out, divergent regions are shown in solid colour. **d,e**, CryoEM data statistics for *ops*PEC (**d**) and nc-*ops*PEC (**e**). The local resolutions (top left), Fourier shell correlation (FSC, top right) and particle angular distributions plots after NU refinement (bottom) are displayed. Local resolutions range from 2.5 Å (blue) to 8.5 Å (red); low resolution regions mainly reside within the upstream DNA and flexible RNAP domains (e.g. SI2 or SI3). **f-h**, Structural comparison of *ops*PEC and nc-*ops*PEC. Top: Models of *ops*PEC (**f**), nc-*ops*PEC (**g**) and their superposition (**h**) are shown as cartoon along with their cryoEM maps (**f** and **g**; transparent surface). RNAP and corresponding maps are colored according to RNAP subunits, nucleic acid density is light blue (**f** and **g**). Bottom: Nucleic acid scaffolds of *ops*PEC (**f**) or nc-*ops*PEC (**g**) together with their cryoEM densities (transparent surface), colored according to local resolution ranging from 2.5 Å (blue) to 8.5 Å (red) and their superposition (**h**). In (**f**) and (**g**), the helix axis vectors of up- and downstream DNA and the angle between them are shown. In (**h**), structures are colored as indicated.

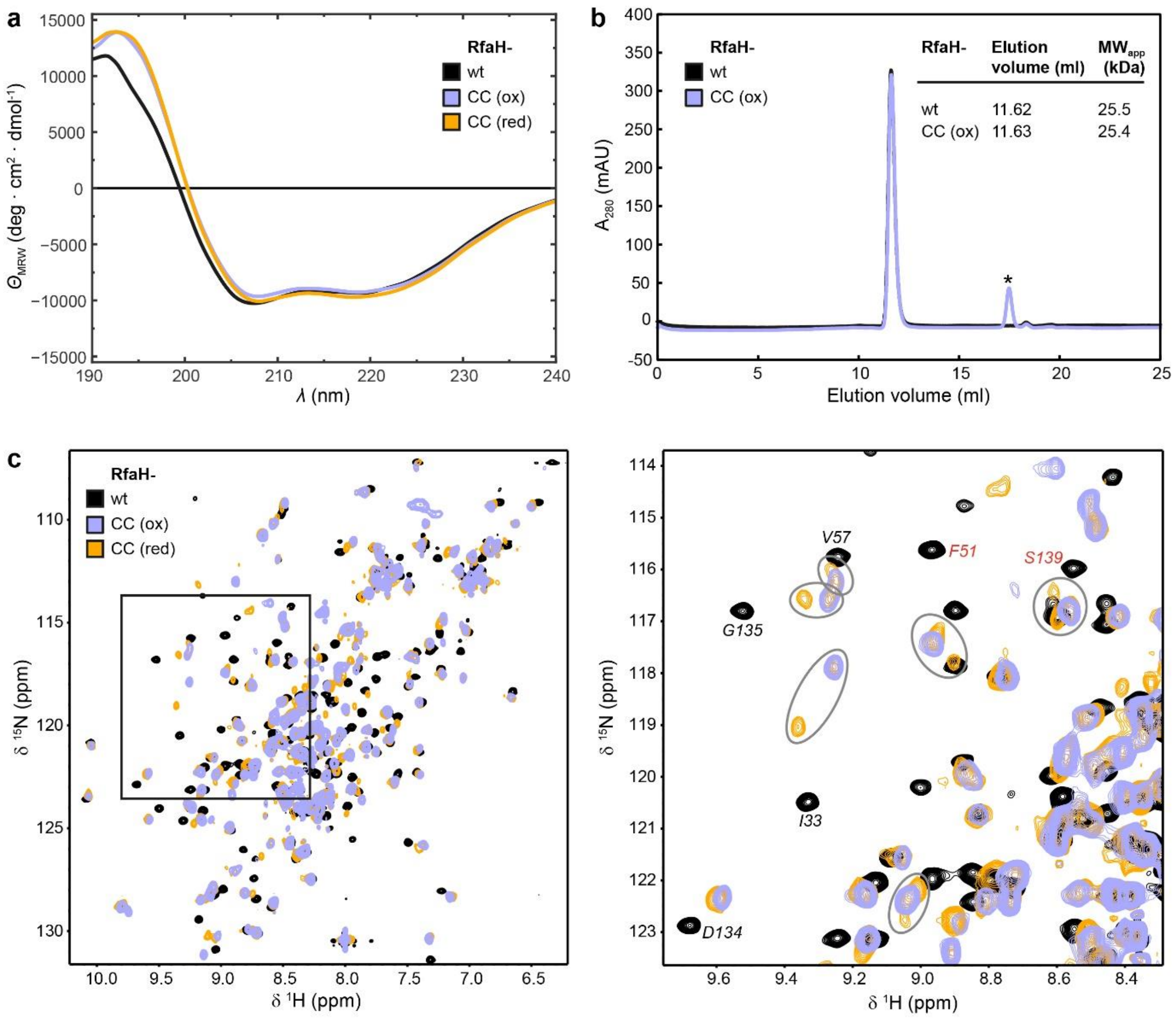

Supplementary Figure 3. Protein quality control of RfaH^{CC}. a, Overlay of normalized CD-spectra of wt RfaH (black) and oxidized RfaH^{CC} (purple), recorded in CD buffer (10 mM KP_i (pH 7.0)) and of RfaH^{CC} in CD buffer containing 0.5 mM TCEP (orange). **b**, Overlay of chromatograms of analytical SEC runs of wt RfaH (black) and oxidized RfaH^{CC} (purple) on a 24 ml Superdex 75 column. The asterisk marks a buffer artefact. The inset shows the elution volumes determined for the main peaks and the proteins' apparent molecular weights calculated by comparison of the elution volumes of standard proteins (see STAR Methods). **c**, Left: Overlay of 2D [^{1}H, ^{15}N]-HSQC spectra of ^{15}N-labelled wt RfaH (black) and RfaH^{CC} in the presence of Cu^{II} phenanthroline (oxidized state; purple) or DTT (reduced state; orange), respectively. Right: Enlargement of the boxed spectral window of the full spectrum (left). Resonances of the original RfaH-F51 and RfaH-S139 backbone amides disappearing due to

the substitutions and of those in spatial proximity to the substitution sites are labelled in red or black, respectively. Ellipses highlight signals that are split in two or more (weak) peaks in the reduced state (indicative of multiple different conformations) but reduce to one (strong) signal in the oxidized state (indicative of fixing one particular conformation by cystine formation).

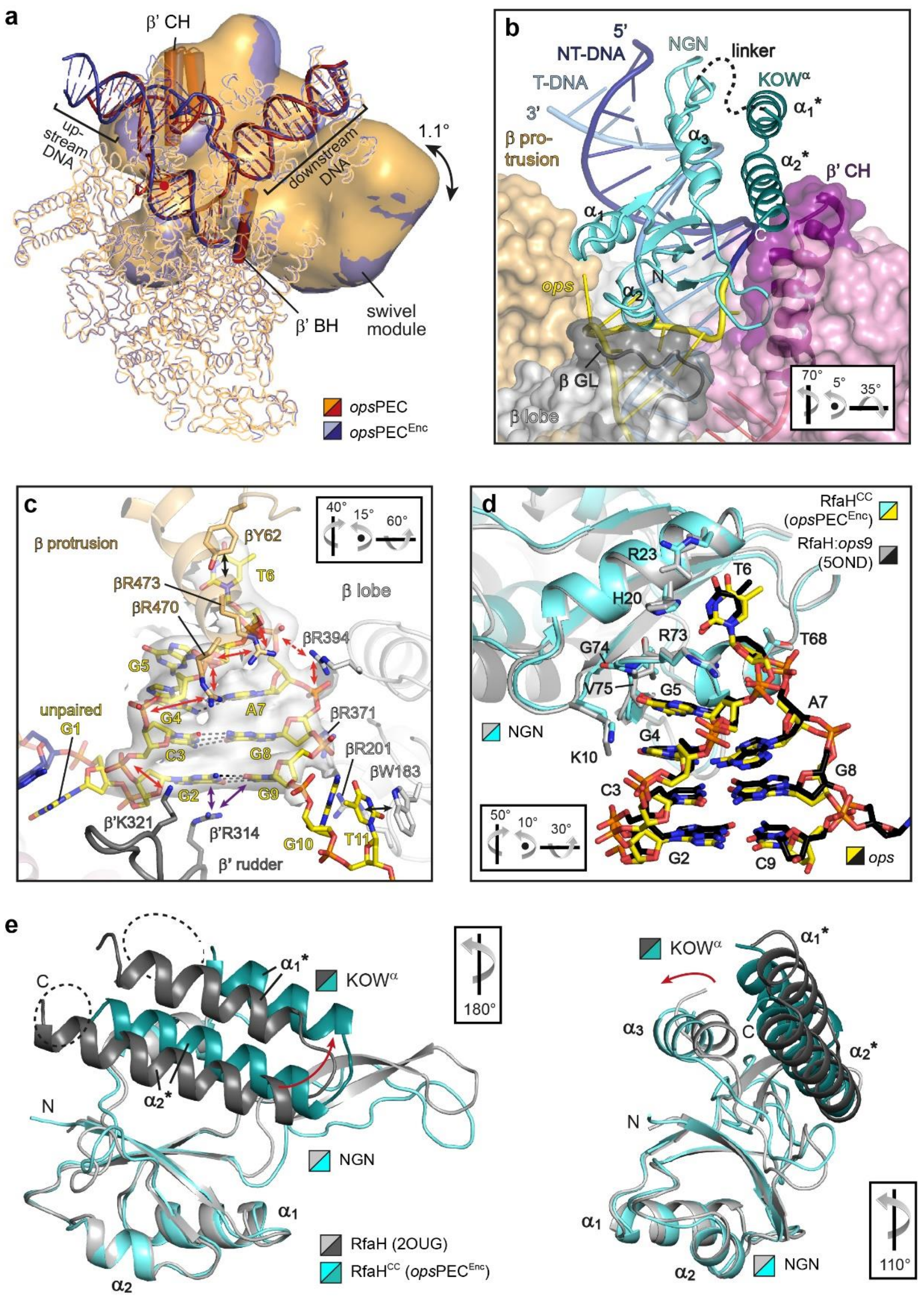

Supplementary Figure 4. Structural analysis and comparison of the *ops*PEC^{Enc} complex. a, *ops*PEC^{Enc} is less swivelled than *ops*PEC. Shown is a structural overlay of the *ops*PEC and *ops*PEC^{Enc}. The two models are superimposed on the core region (ribbons) with the swivel module being represented as Gaussian surface. Swivel angle and approximate axis

(red dot) are indicated. The β' BH and β' CH are shown as cartoon tubes to indicate the orientation of the swivelling axis. **b**, The RfaH^{CC} KOW$^{\alpha}$ domain is located on top of the β' CH. RNAP is depicted as molecular surface; selected structural elements are shown as cartoons and coloured as indicated. RfaH^{CC} (cyan/mint) is in cartoon representation, relevant secondary structure elements are labelled. The orientation relative to the standard view (Fig. 2a) is indicated. **c**, Accommodation of the *ops*HP within the *ops*PEC^{Enc} complex. *ops* DNA is in stick representation, selected RNAP elements are in cartoon with side chains of residues contacting *ops* shown as sticks. H-bonds of the *ops*HP base pairs are represented by dashed lines, stacking interaction by arrows. The CryoEM map of the *ops*HP is shown as transparent surface. The orientation relative to the standard view (Fig. 2a) is indicated. **d**, Recognition of *ops* by RfaH in *ops*PEC^{Enc} is the same as in bimolecular RfaH:*ops* complex. Superposition of the RfaH:*ops*9 complex of a co-crystal structure (PDB-ID: 5OND) and RfaH^{CC}:*ops*HP of the *ops*PEC^{Enc} complex. RfaH in cartoon representation, side chains of *ops* interacting residues and *ops* DNAs shown as sticks. Colors are as indicated. The orientation relative to the standard view (Fig. 2a) is indicated. **e**, The RfaH^{CC} KOW$^{\alpha}$ helices shift position upon binding to the *ops*PEC and unfold partially. Overlay of free RfaH (PDB-ID: 2OUG, cyan/mint) and RfaH^{CC} of the *ops*PEC^{Enc} (light/dark gray). Alpha helices and termini are labelled. Left: Shift of the KOW$^{\alpha}$ helices away from the NGN, and the unfolding of the C-terminal α_1* and α_2* helix turns. Right: NGN helix α_3 is pushed together with the KOW$^{\alpha}$. The orientations relative to the standard view (Fig. 2a) are indicated.

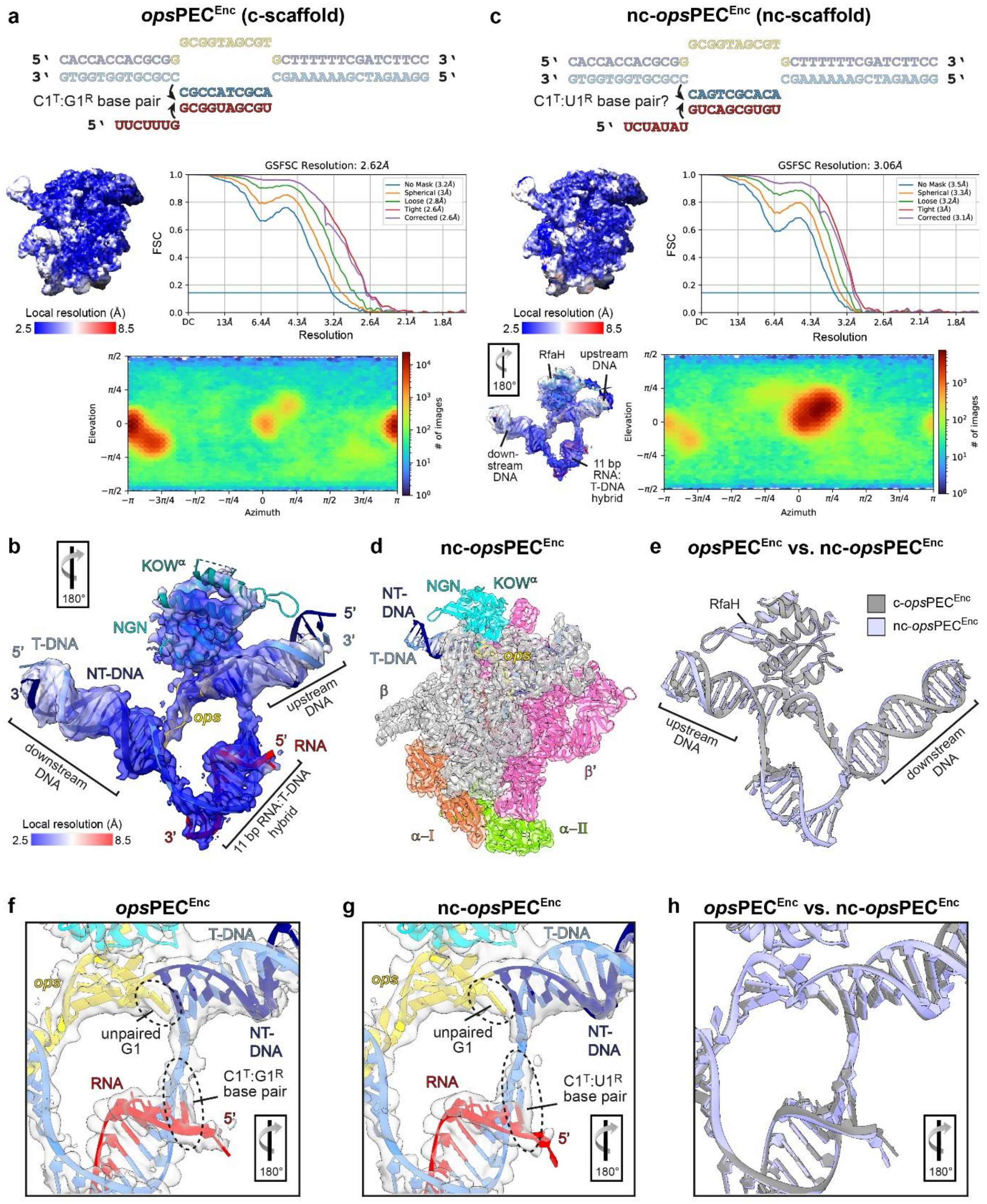

Supplementary Figure 5. Comparison of the *ops*PEC^{Enc} and nc-*ops*PEC^{Enc} structures. **a,c**, Top: Nucleic acids scaffolds used for reconstitution of the *ops*PEC^{Enc} (**a**) or nc-*ops*PEC^{Enc} complexes (**c**). Regions identical in both scaffolds are grayed out, divergent regions are shown in solid color. The arrows show formation of the C1^{T}:G1^{R} base pair in *ops*PEC upon binding of RfaH^{CC} (**a**), or the formation of a non-canonical C1^{T}:U1^{R} base pair in the non-

complementary scaffold (**c**). Middle left: Local resolutions of the respective complexes range from 2.5 Å (blue) to 8.5 Å (red); low resolution regions mainly reside within the upstream DNA and flexible RNAP domains (e.g., SI2 or SI3). Middle right: Fourier shell correlation plots of the two complexes. Bottom (**a**) and bottom right (**c**): Particle angular distribution plots. Bottom left (**c**): Nucleic acid scaffolds of nc-*ops*PEC together with its cryoEM density (transparent surface), colored according to local resolution, ranging from 2.5 Å (blue) to 8.5 Å (red). **b**, nucleic acid scaffold of *ops*PEC^{Enc} together with its cryoEM density (transparent surface), colored according to local resolution, ranging from 2.5 Å (blue) to 8.5 Å (red). **d,** CryoEM map (transparent surface) and model (in cartoon representation) of the nc-*ops*PEC^{Enc} complex. Colors as indicated. **e**, Overlay of *ops*PEC^{Enc} and nc-*ops*PEC^{Enc} nucleic acids and RfaH^{CC} structures. Models are shown as cartoon with colors as indicated. **f-h**, Close-up views of the upstream edge of the transcription bubbles in *ops*PEC^{Enc} and nc-*ops*PEC^{Enc}. Nucleic acid scaffolds (cartoon representation) and their associated cryoEM densities (transparent surfaces) are shown for *ops*PEC^{Enc} (**f**), nc-*ops*PEC^{Enc} (**g**) and an overlay of the two structures (**h**). The G1 base is unpaired in both structures and a 11th RNA:T-DNA hybrid base pair at its upstream end is formed instead (C1^{T}:G1^{R} in *ops*PEC^{Enc} and a non-canonical C1^{T}:U1^{R} in nc-*ops*PEC^{Enc}). The orientations relative to the standard view (Fig. 2a) are indicated.

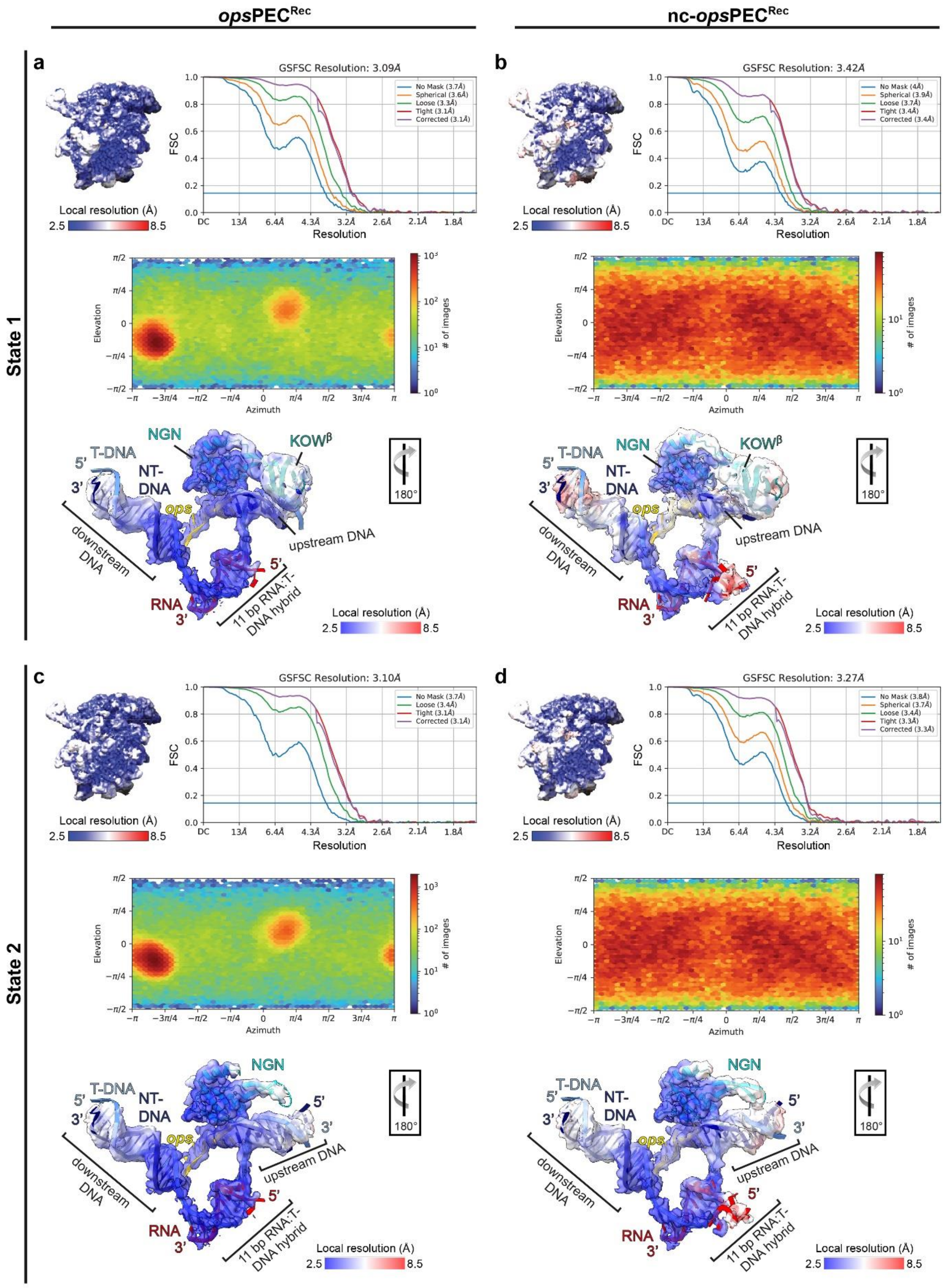

Supplementary Figure 6. Analysis of the cryoEM data sets of *ops*PEC^{Rec} and nc-*ops*PEC^{Rec} states 1 and 2. a-d, Top left: Local resolutions of the complexes plotted on their corresponding cryoEM densities. The local resolutions range from 2.5 Å (blue) to 8.5 Å (red).

Low resolution regions mainly reside within the upstream DNA and flexible RNAP domains. Top right: Fourier shell correlation plots. Middle: Particle angular distribution plots of the corresponding complexes. Bottom: Models of nucleic acid scaffolds and RfaH together with their cryoEM densities (transparent surface), colored according to local resolution ranging from 2.5 Å (blue) to 8.5 Å (red). The orientations relative to the standard view (Fig. 2a) are indicated. States 1 and 2 represent the two states obtained from 3DVA of the *ops*PEC^{Rec} or nc-*ops*PEC^{Rec} complexes, respectively.

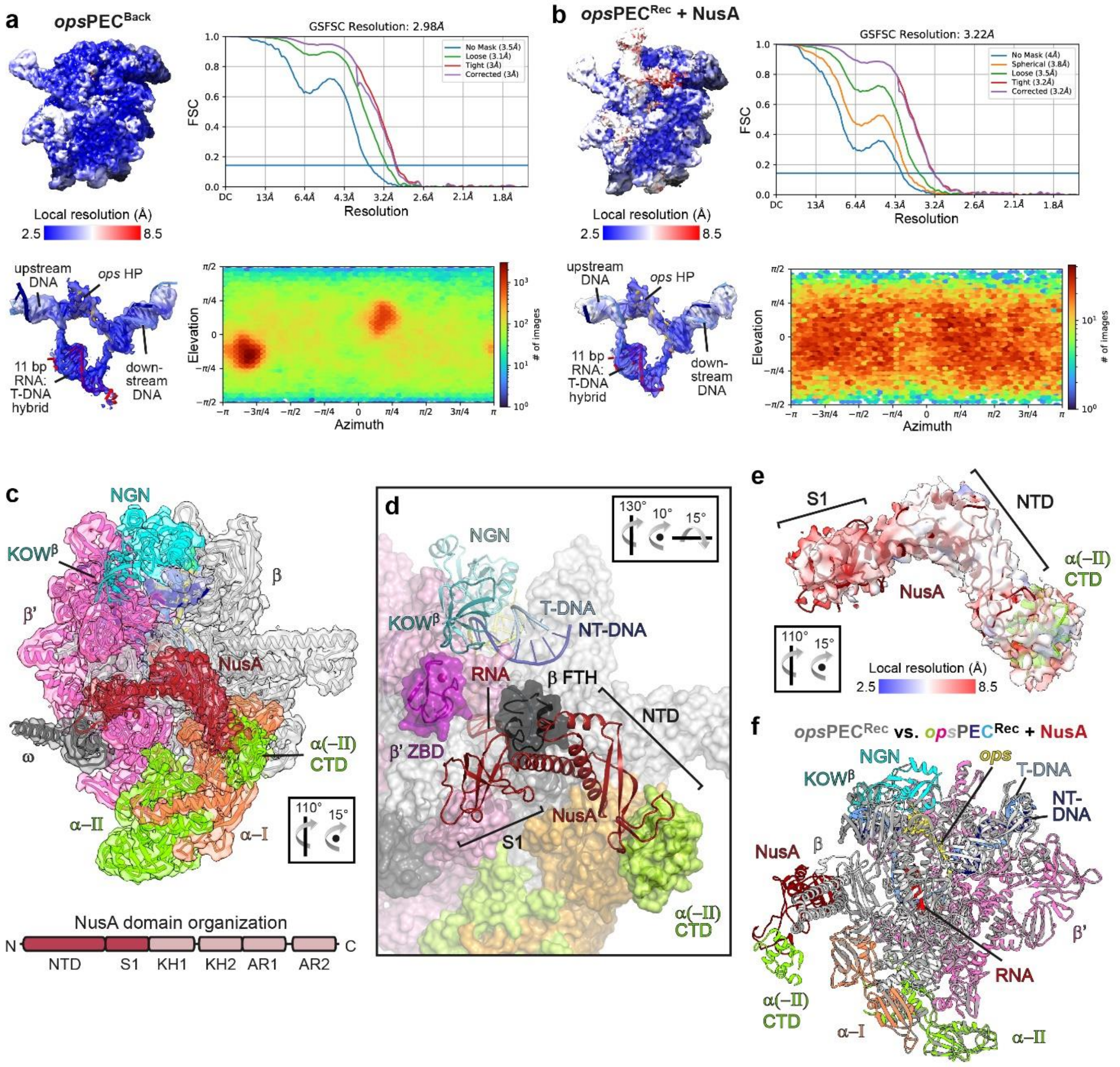

Supplementary Figure 7. CryoEM data and map quality parameters of the *ops*PEC^{Back} and *ops*PEC^{Rec}:NusA complexes, and structural details of the NusA modified complex. **a,b**, Left: Local resolutions of the *ops*PEC^{Back} (**a**) or *ops*PEC^{Rec} + NusA (**b**) complexes plotted on the corresponding cryoEM densities of the complete complex (top), or the nucleic acid scaffold (nucleic acids show as cartoon). Local resolutions range from 2.5 Å (blue) to 8.5 Å (red). Low resolution regions mainly reside within the upstream DNA and flexible RNAP domains. Middle: Fourier shell correlation plots. Right: Particle angular distribution plots of the corresponding complexes. **c,** Overview of the *ops*PEC^{Rec} + NusA complex. The cryoEM map of the complex is shown along with the model, both color-coded. The domain organization of NusA is depicted at the bottom; grayed-out domains are not resolved in the map, i.e. only

NusA-NTD and S1 domains are visible. The orientation relative to the standard view (Fig. 2a) is indicated. **d**, Close-up view of the NusA:RNAP interactions. RNAP is in surface representation, domains interacting with either NusA (βFTH and αCTD) or RfaH KOW^{β} domain (β' ZBD) are shown as cartoon (the Zn^{2+} ion of the β' ZBD is shown as sphere). NusA is displayed in cartoon representation, domains are labelled. The orientation relative to the standard view (Fig. 2a) is indicated. **e**, Cartoon representation of NusA and αCTD within *ops*PEC^{Rec} + NusA complex with their associated cryoEM densities colored according to local resolution ranging from 2.5 Å (blue) to 8.5 Å (red). **f**, NusA binding to the *ops*PEC^{Rec} does not alter the conformation of the RNAP, nucleic acids or RfaH. Superposition of the *ops*PEC^{Rec} (gray) and the *ops*PEC^{Rec} + NusA complexes (colored as indicated), both depicted in cartoon representation.

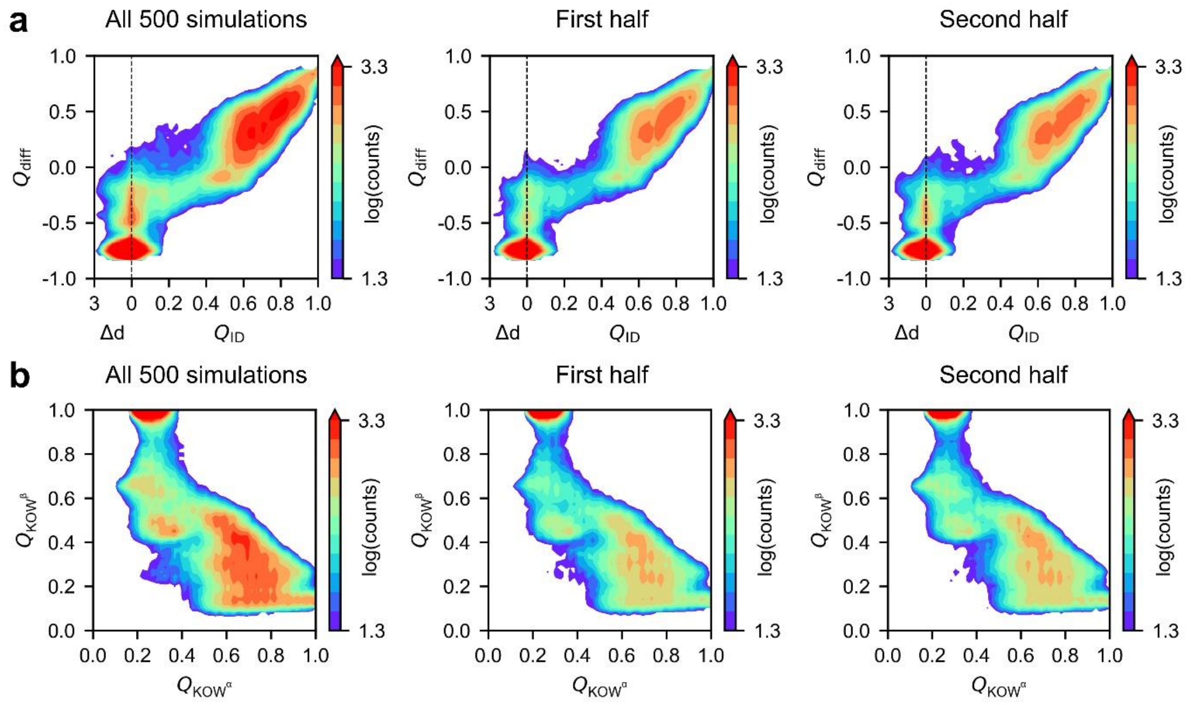

Supplementary Figure 8. Re-analysis of the simulation data split in two halves demonstrates sufficient sampling. To demonstrate sufficient sampling of the RfaH refolding process in the context of the *ops*PEC complex, additional refolding landscapes were calculated after splitting the resulting data from refolding simulations in two halves. **a**, Refolding landscapes are projected onto Q_{ID} (fraction of ID contacts), Δd (distance between domains with respect to the distance in the active state, in nm) and Q_{diff} (difference in native contacts between the KOW^{α} and KOW^{β}). **b**, Refolding landscapes are projected onto the fraction of native contacts of each KOW state ($Q_{KOW^{\alpha}}$ and $Q_{KOW^{\beta}}$).

Supplementary References

1. Kang, J.Y. et al. RNA Polymerase Accommodates a Pause RNA Hairpin by Global Conformational Rearrangements that Prolong Pausing. *Mol Cell* **69**, 802-815 e805 (2018).
2. Guo, X. et al. Structural Basis for NusA Stabilized Transcriptional Pausing. *Mol Cell* **69**, 816-827 e814 (2018).
3. Kang, J.Y. et al. An ensemble of interconverting conformations of the elemental paused transcription complex creates regulatory options. *Proc Natl Acad Sci U S A* **120**, e2215945120 (2023).
4. Vvedenskaya, I.O. et al. Interactions between RNA polymerase and the "core recognition element" counteract pausing. *Science* **344**, 1285-1289 (2014).
5. Larson, M.H. et al. A pause sequence enriched at translation start sites drives transcription dynamics in vivo. *Science* **344**, 1042-1047 (2014).
6. Artsimovitch, I. & Landick, R. The transcriptional regulator RfaH stimulates RNA chain synthesis after recruitment to elongation complexes by the exposed nontemplate DNA strand. *Cell* **109**, 193-203 (2002).
7. Svetlov, V. & Artsimovitch, I. Purification of bacterial RNA polymerase: tools and protocols. *Methods Mol Biol* **1276**, 13-29 (2015).
8. Artsimovitch, I. et al. Allosteric modulation of the RNA polymerase catalytic reaction is an essential component of transcription control by rifamycins. *Cell* **122**, 351-363 (2005).
9. Belogurov, G.A. et al. Structural basis for converting a general transcription factor into an operon-specific virulence regulator. *Mol Cell* **26**, 117-129 (2007).
10. Deaconescu, A.M. & Darst, S.A. Crystallization and preliminary structure determination of Escherichia coli Mfd, the transcription-repair coupling factor. *Acta Crystallogr F* **61**, 1062-1064 (2005).
11. Strauss, M. et al. Transcription is regulated by NusA:NusG interaction. *Nucleic Acids Res* **44**, 5971-5982 (2016).
12. Zuber, P.K., Schweimer, K., Rosch, P., Artsimovitch, I. & Knauer, S.H. Reversible fold-switching controls the functional cycle of the antitermination factor RfaH. *Nat Commun* **10**, 702 (2019).
