# Supplementary material for: Concerted transformation of a hyper-paused transcription complex and its reinforcing protein
Source: Nat Commun. 2024 Apr 8;15:3040. doi: 10.1038/s41467-024-47368-4 (PMC11001881; doi:10.1038/s41467-024-47368-4)
Supplement: Supplementary file 3 — Description of Additional Supplementary Files [file 41467_2024_47368_MOESM3_ESM.pdf]

Description of Additional Supplementary Files

File name: Supplementary Movie 1
Description: RfaH refolding trajectory 1. RfaH refolding follows heterogeneous trajectories. The movie displays RfaH refolding trajectory 1 (β1*-β5* order).

File name: Supplementary Movie 2
Description: RfaH refolding trajectory 2. RfaH refolding follows heterogeneous trajectories. The movie displays RfaH refolding trajectory 2 (β1*-β2* order).

File name: Supplementary Movie 3
Description: 3DVA of *ops*PEC^{Rec} - KOW^{β}/upstream DNA. The movie shows how in *ops*PEC^{Rec}, KOW^{β} binding at the β'ZBD is correlated with a movement of the upstream DNA towards the β protrusion and flap.

File name: Supplementary Movie 4
Description: 3DVA of *ops*PEC^{Rec} - KOW^{β}/*ops*HP. The movie shows how in *ops*PEC^{Rec}, density for KOW^{β} is anti-correlated with density for the *ops*HP.
