# Supplementary material for: Concerted transformation of a hyper-paused transcription complex and its reinforcing protein
Source: Nat Commun. 2024 Apr 8;15:3040. doi: 10.1038/s41467-024-47368-4 (PMC11001881; doi:10.1038/s41467-024-47368-4)
Supplement: Supplementary file 8 — Reporting Summary [file 41467_2024_47368_MOESM8_ESM.pdf]

nature portfolio

Corresponding author(s): Irina Artsimovitch, Markus C. Wahl, Stefan H. Knauer

Last updated by author(s): Mar 10, 2024

Reporting Summary

Nature Portfolio wishes to improve the reproducibility of the work that we publish. This form provides structure for consistency and transparency in reporting. For further information on Nature Portfolio policies, see our Editorial Policies and the Editorial Policy Checklist.

Statistics

For all statistical analyses, confirm that the following items are present in the figure legend, table legend, main text, or Methods section.

n/a	Confirmed	
☐	☒	The exact sample size (*n*) for each experimental group/condition, given as a discrete number and unit of measurement
☐	☒	A statement on whether measurements were taken from distinct samples or whether the same sample was measured repeatedly
☒	☐	The statistical test(s) used AND whether they are one- or two-sided<br>*Only common tests should be described solely by name; describe more complex techniques in the Methods section.*
☒	☐	A description of all covariates tested
☒	☐	A description of any assumptions or corrections, such as tests of normality and adjustment for multiple comparisons
☐	☒	A full description of the statistical parameters including central tendency (e.g. means) or other basic estimates (e.g. regression coefficient) AND variation (e.g. standard deviation) or associated estimates of uncertainty (e.g. confidence intervals)
☒	☐	For null hypothesis testing, the test statistic (e.g. *F*, *t*, *r*) with confidence intervals, effect sizes, degrees of freedom and *P* value noted<br>*Give P values as exact values whenever suitable.*
☒	☐	For Bayesian analysis, information on the choice of priors and Markov chain Monte Carlo settings
☒	☐	For hierarchical and complex designs, identification of the appropriate level for tests and full reporting of outcomes
☒	☐	Estimates of effect sizes (e.g. Cohen's *d*, Pearson's *r*), indicating how they were calculated

Our web collection on statistics for biologists contains articles on many of the points above.

Software and code

Policy information about availability of computer code

Data collection	EPU, version 2.8.1 (cryoEM/SPA data acquisition; Thermo Fisher Scientific; referenced in text)
Data analysis	ChimeraX, version 1.7 (cryoEM reconstructions figure preparation; referenced in text)<br>COOT, version 0.9.8.1 (model building; referenced in text)<br>cryoSPARC, version 3.2-4.0.2 (all cryoEM image analysis; referenced in text)<br>GROMACS, version 4.5 (energy minimization in explicit solvent; referenced in text)<br>ImageQuant, version 5.2 (quantification of bands on gels; referenced in text)<br>Modeller, version 10.4 (adding missing residues to experimental models; referenced in text)<br>MolProbity, version 4.5.1 (structure evaluation; referenced in text)<br>NMRViewJ, version 9.2.b20 (analysis of HSQC spectra; referenced in text)<br>PHENIX, version 1.20_44591 (real space refinement; referenced in text)<br>PyMOL, version 2.5.0 (modeling, model superpositioning and extraction of model geometric parameters; referenced in text)<br>SMOG 2, version 2.4.5 (generation of all-atom structure-based models; references in text)<br>TopSpin, version 3.5 (acquisition of NMR spectra)

For manuscripts utilizing custom algorithms or software that are central to the research but not yet described in published literature, software must be made available to editors and reviewers. We strongly encourage code deposition in a community repository (e.g. GitHub). See the Nature Portfolio guidelines for submitting code & software for further information.

Data

Policy information about availability of data

All manuscripts must include a data availability statement. This statement should provide the following information, where applicable:

- Accession codes, unique identifiers, or web links for publicly available datasets
- A description of any restrictions on data availability
- For clinical datasets or third party data, please ensure that the statement adheres to our policy

CryoEM reconstructions have been deposited in the Electron Microscopy Data Bank (https://www.ebi.ac.uk/pdbe/emdb) under accession codes EMD-17626 [https://www.ebi.ac.uk/pdbe/entry/emdb/EMD-17626] (opsPEC), EMD-17657 [https://www.ebi.ac.uk/pdbe/entry/emdb/EMD-17657] (nc-opsPEC), EMD-17679 [https://www.ebi.ac.uk/pdbe/entry/emdb/EMD-17679] (opsPECEnc), EMD-17646 [https://www.ebi.ac.uk/pdbe/entry/emdb/EMD-17646] (nc-opsPECEnc), EMD-17668 [https://www.ebi.ac.uk/pdbe/entry/emdb/EMD-17668] (opsPECRec state 1), EMD-17632 [https://www.ebi.ac.uk/pdbe/entry/emdb/EMD-17632] (opsPECRec state 2), EMD-17686 [https://www.ebi.ac.uk/pdbe/entry/emdb/EMD-17686] (nc-opsPECRec state 1), EMD-17647 [https://www.ebi.ac.uk/pdbe/entry/emdb/EMD-17647] (nc-opsPECRec state 2), EMD-17681 [https://www.ebi.ac.uk/pdbe/entry/emdb/EMD-17681] (opsPECback), and EMD-17685 [https://www.ebi.ac.uk/pdbe/entry/emdb/EMD-17685] (opsPECRec,NusA). Structure coordinates have been deposited in the RCSB Protein Data Bank (https://www.rcsb.org) with accession codes 8PDY [https://www.rcsb.org/structure/8PDY] (opsPEC), 8PH9 [https://www.rcsb.org/structure/8PH9] (nc-opsPEC), 8PIB [https://www.rcsb.org/structure/8PIB] (opsPECEnc), 8PFG [https://www.rcsb.org/structure/8PFG] (nc-opsPECEnc), 8PHK [https://www.rcsb.org/structure/8PHK] (opsPECRec state 1), 8PEN [https://www.rcsb.org/structure/8PEN] (opsPECRec state 2), 8PIM [https://www.rcsb.org/structure/8PIM] (nc-opsPECRec state 1), 8PFJ [https://www.rcsb.org/structure/8PFJ] (nc-opsPECRec state 2), 8PID [https://www.rcsb.org/structure/8PID] (opsPECback), and 8PIL [https://www.rcsb.org/structure/8PIL] (opsPECRec,NusA). All-atom structure-based models of opsPECEnc and opsPECRec, representative structures for intermediates retrieved from 500 simulations of RfaH refolding in the absence and presence of opsPEC, and representative trajectories of RfaH fold-switching while bound to opsPEC, are available at Zenodo [https://doi.org/10.5281/zenodo.10493315]. All other data are contained in the manuscript or the Supplementary Information. Source data are provided with this paper. Structure coordinates used in this study are available from the RCSB Protein Data Bank (https://www.rcsb.org) under accession codes 2OUG [https://www.rcsb.org/structure/2OUG], 5OND [https://www.rcsb.org/structure/5OND], 5VOI [https://www.rcsb.org/structure/5VOI], 6ALF [https://www.rcsb.org/structure/6ALF], 6C6S [https://www.rcsb.org/structure/6C6S], 6RH3 [https://www.rcsb.org/structure/6RH3], 7YPA [https://www.rcsb.org/structure/7YPA], 8EG7 [https://www.rcsb.org/structure/8EG7], 8EG8 [https://www.rcsb.org/structure/8EG8], 8EH8 [https://www.rcsb.org/structure/8EH8], and 8FVW [https://www.rcsb.org/structure/8FVW].

Research involving human participants, their data, or biological material

Policy information about studies with human participants or human data. See also policy information about sex, gender (identity/presentation), and sexual orientation and race, ethnicity and racism.

Reporting on sex and gender	n.a.
Reporting on race, ethnicity, or other socially relevant groupings	n.a.
Population characteristics	n.a.
Recruitment	n.a.
Ethics oversight	n.a.

Note that full information on the approval of the study protocol must also be provided in the manuscript.

Field-specific reporting

Please select the one below that is the best fit for your research. If you are not sure, read the appropriate sections before making your selection.

☒ Life sciences ☐ Behavioural & social sciences ☐ Ecological, evolutionary & environmental sciences

For a reference copy of the document with all sections, see nature.com/documents/nr-reporting-summary-flat.pdf

Life sciences study design

All studies must disclose on these points even when the disclosure is negative.

Sample size	For cryoEM analyses, sample sizes were chosen to yield a large number of particle images on the grids while avoiding non-specific aggregation. The number of particle images was chosen to yield cryoEM reconstructions at sufficient resolution for reliable fitting and refinement of atomic models. Numbers of particle images used for reconstructions are listed in Supplementary Tables 1 and 2.<br>For in vitro transcription assays, sample sizes were chosen to minimize pipetting errors and to provide clearly visible and quantifiable bands on gels. No calculations were performed to select the sample size as such calculations are unnecessary; these are bulk assays, with more than one billion molecules of active transcription elongation complexes and accessory factors (when added) present in each sample.
Data exclusions	Structural analysis by cryoEM involved the sorting of high quality particle images and rejection of poor quality particle images. Poor quality particle images may be due to compositional heterogeneity in the sample or may originate from particles being damaged in the process of

grid preparation, e.g. at the air-water interface. The processing steps involving data exclusion are outlined in Supplementary Figure 1. For other experiments, no data were excluded from the analyses.

Replication	CryoEM-based structural analyses were conducted once for each complex.<br>In vitro transcription assays were carried out at least in triplicates. All attempts at replication were successful, as is typical for such assays.<br>A total of 500 MD simulations were run per simulation system.
Randomization	All MD simulations were provided different random seeds.<br>For other experiments, randomization was not required as (1) no human or animal subjects were studied, (2) quantitative data were collected, (3) no subjective interpretations were required and (4) there was no danger of confounding independent variables in the experimental design.
Blinding	Blinding is not applicable to experiments reported herein, as the experiments did not involve human or animal subjects, and as the results from the experiments can be objectively evaluated/quantified.

Reporting for specific materials, systems and methods

We require information from authors about some types of materials, experimental systems and methods used in many studies. Here, indicate whether each material, system or method listed is relevant to your study. If you are not sure if a list item applies to your research, read the appropriate section before selecting a response.

Materials & experimental systems

n/a	Involved in the study
☒	☐ Antibodies
☒	☐ Eukaryotic cell lines
☒	☐ Palaeontology and archaeology
☒	☐ Animals and other organisms
☒	☐ Clinical data
☒	☐ Dual use research of concern
☒	☐ Plants

Methods

n/a	Involved in the study
☒	☐ ChIP-seq
☒	☐ Flow cytometry
☒	☐ MRI-based neuroimaging

Plants

Seed stocks	n.a.
Novel plant genotypes	n.a.
Authentication	n.a.
